# Patients with Higher Pulse Wave Velocity Are More Likely to Develop a More Severe Form of Knee Osteoarthritis: Implications for Cardiovascular Risk

**DOI:** 10.3390/biomedicines13051208

**Published:** 2025-05-15

**Authors:** Tina Zavidić, Emina Babarović, Vedrana Drvar, Božena Ćurko-Cofek, Gordana Laškarin

**Affiliations:** 1 Department of Family Medicine, Faculty of Medicine, University of Rijeka, B. Branchetta 20, 51000 Rijeka, Croatia; 2 Istrian Helath Centers, J. Dobrile 1, 52000 Pazin, Croatia; 3 Department of Pathology, Faculty of Medicine, University of Rijeka, B. Branchetta 20, 51000 Rijeka, Croatia; emina.babarovic@uniri.hr; 4 Clinical Institute of Laboratory Diagnostics, University Hospital Centre Rijeka, V. Dukića 7, 51000 Rijeka, Croatia; vedranadrvar@gmail.com; 5 Department of Physiology, Immunology and Pathophysiology, Faculty of Medicine, University of Rijeka, B. Branchetta 20, 51000 Rijeka, Croatia; bozena.curko.cofek@uniri.hr (B.Ć.-C.); gordana.laskarin@uniri.hr (G.L.); 6 Hospital for Medical Rehabilitation of the Heart and Lung Diseases and Rheumatism “Thalassotherapia Opatija”, M. Tita 188, 51412 Opatija, Croatia

**Keywords:** arterial stiffness, cardiovascular risk, knee, osteoarthritis, pulse wave velocity

## Abstract

**Background/Objectives**: Knee osteoarthritis (KOA) is a progressive degenerative joint disease characterised by low-grade inflammation and is associated with increased cardiovascular (CV) risk and arterial stiffness. Pulse wave velocity (PWV) is a quantitative measure of arterial stiffness and an important tool for detecting subclinical arterial calcification and CV risk. This study aimed to determine whether PWV can distinguish radiographically mild KOA (Kellgren–Lawrence grades 1-2) from severe KAO (Kellgren–Lawrence grades 3-4) in terms of CV risk factors. **Methods**: A total of 223 postmenopausal women with KOA participated in this cross-sectional study. Assessments included anthropometry, laboratory analyses, blood pressure and PWV measurements, a 6 min walk test, pain evaluation using a visual analogue scale (VAS), and completion of the International Physical Activity Questionnaire (IPAQ). **Results**: PWV was significantly higher in the severe KOA group (10.53 m/s vs. 8.78 m/s, *p* < 0.001). A cut-off value of 8.4 m/s effectively distinguished between severe and mild forms of KOA (AUC = 0.798, *p* = 0.001). OA grade, pain, age, waist circumference, WHR, SCORE 2/SCORE 2OP, systolic blood pressure, serum glucose, HbA1c, uric acid, creatinine, and erythrocyte sedimentation rate were increased in the group with PWV > 8.4 m/s, compared to the group with PWV ≤ 8.4 m/s. Conversely, eGFR, the 6 min walk test and physical activity of patients were reduced in the group with PWV > 8.4 m/s. A patient with a PWV > 8.4 m/s has a 1.77 times higher chance of developing a more severe form of the disease than a patient with a lower PWV. **Conclusions**: Patients with a higher PWV are more likely to develop a more severe form of KOA, which is associated with increased cardiovascular risk.

## 1. Introduction

Osteoarthritis (OA) is the most common debilitating joint disease, and in 2020, nearly 600 million people worldwide were registered with OA, with a prevalence of approximately 18% in the population between 30 and 60 years of age [1]. The prevalence is twice as high in women as in men [2]. The knee is the most common site of OA due to its anatomical structure and physiological mechanical load [3,4]. Therefore, knee OA (KOA) significantly reduces functional capacity [4] and is estimated to affect approximately 365 million registered patients, with different prevalence in different regions [3]. Globally, KOA mainly affects obese women with lower levels of education, and as body mass index increases, so does the risk of developing KOA [1]. Two-thirds of obese adults develop symptomatic OA [5], and approximately 70% of all knee replacements are attributed to obesity [6]. Alongside excessive joint weight loading, adipokines secreted by white adipose tissue support the development of KOA before clinical manifestations [7]. Leptin, a precursor of adipokines, has been associated with the development of KOA, and its elevation in serum levels of 5 μg/L is associated with a 30% increased risk of structural KOA in obese patients [8].

On the other hand, damage to joint structures, as a result of a strong impact, increases the risk of KOA, especially rupture of the anterior cruciate ligament and injury to the meniscus of the knee [9]. Therefore, OA was long thought to be caused by simple joint “wear and tear”, resulting from mechanically damaged chondrocytes, which prevent cartilage repair. However, limited inflammatory changes were also recognised at the cartilage–synovial membrane junctions early in the disease [10]. Fragments from damaged cartilage represent disease-associated molecular patterns, which are released into the extracellular space and synovial fluid in response to damage, as endogenous danger signals and initiate inflammation and pain via TLR signaling [11]. Local hyperplasia of the synovial membrane with angiogenesis and leukocyte infiltration is recognised as a sign of acute low-grade inflammation, with occasional exacerbations in the vicinity of mechanical cartilage and bone load [12]. Over time, soluble components of progressively damaged cartilage and subchondral bone induce persistent low-grade synovitis with thickening of the synovial membrane, ligaments, and tendons involving the fibrosis of the infrapatellar fat pad and weakness of the surrounding muscles [13], intensifying the pain, joint stiffness, and functional disability of patients [14]. This is how OA presents a chronic progressive disease of all the tissues that make up the joint with possible impacts on the health of the entire organism [15], especially during periods of pain mediated by inflammatory substances [16].

Pro-inflammatory mediators and proteolytic enzymes that have entered the circulation from OA joints [16,17] start to disintegrate the endothelial glycocalyx [18], together with products of cytokine-activated circulating leukocytes [19], creating the conditions for endothelial dysfunction and arterial stiffness [20]. It appears that the rate of arterial stiffness progression in OA patients may be driven and accelerated by various factors, including the interaction of mechanical, inflammatory, and metabolic stimuli [9].

Cardiovascular comorbidities based on endothelial dysfunction cause irreversible blood vessel stiffness over time [21]. Medial calcification is responsible for medium-sized arterial stiffness [22], whereas intimal calcification is associated with atherosclerosis of the aorta and large arteries [23]. Arterial stiffness amplifies and accelerates wave reflections at the level of the aorta, widens pulse pressure, increases afterload and systolic hypertension, reduces coronary perfusion, impairs diastolic function, and causes heart failure with preserved ejection fraction [23,24]. Intimal calcification of large arteries is associated with atherosclerosis, arterial obstruction and plaque rupture, often leading to acute myocardial infarction or cerebrovascular events [24,25], in addition to arterial wall stiffness [23]. Carotid–femoral pulse wave velocity (PWV) is a measure of aortic stiffness [26], while brachial–ankle PWV measures large- and medium-sized arterial stiffness of the lower limbs and is an independent vascular biomarker of CV morbidity and mortality [23]. Pulse wave velocity (PWV), an important metric according to recent guidelines on vascular aging, can be measured in several ways [27]. PWV is a widely recognised and independent predictor of cardiovascular events [27]. Both invasive approaches, which are mainly used in research settings, and non-invasive techniques, such as tonometry, oscillometry, ultrasonography, and MRI (magnetic resonance imaging)-based techniques, are included in this group [28]. The gold standard for assessing arterial stiffness is the direct measurement of carotid–femoral PWV (cf-PWV). However, its routine usage in primary care is limited due to a requirement for specialised equipment and trained personnel. To address this limitation, automated oscillometric devices, such as the Agedio^®^B900 device, have been widely used in primary care. These small devices offer completely automated PWV evaluation with minimal operator involvement. Based on upper-arm cuff-based pressure measurements, they reconstruct the aortic pulse waveform and provide an estimated PWV, which has shown a high correlation with cf-PWV. As such, they represent a validated and practical alternative for vascular screening, particularly suited to family medicine and everyday clinical practice [28].

Classical cardiovascular risk factors, all of which cause endothelial dysfunction, such as increased blood pressure (BP), cholesterol, HDL, LDL, triglycerides, glucose, increased body mass index, age, and smoking cigarettes, independently and additively increase arterial stiffness [29]. Recently, inflammation has been recognised as a non-classical risk factor for arterial stiffness in various clinical settings, including OA [19,21,26,30].

This study aimed to determine whether PWV can distinguish the mild form (Kellgren–Lawrence grades 1–2) from the severe form (Kellgren–Lawrence grades 3–4) of KOA in a family doctor’s office in relation to CV risk factors.

## 2. Materials and Methods

### 2.1. Patients

This study was designed as a cross-sectional study, conducted at two family medicine offices of Istrian Health Centers, Croatia, from March 2024 to September 2024. Medical examination consisted of assessing whether the criteria for KOA [31], rheumatoid arthritis [32] and spondyloarthritis [33] were met in order to prove KOA, as well as to exclude a rheumatologic etiology of the knee disease. The applied classification criteria for rheumatoid arthritis [32] are capable of recognising the majority of symptomatic patients who are not yet showing radiographic changes [34], as well as criteria for spondyloarthritis [33], which was of particular interest in this investigation.

Inclusion criteria were as follows:

▪ Postmenopausal women with knee pain;
▪ Provided written informed consent;
▪ Were diagnosed with KOA (X-rays, ultrasound);
▪ Were able to complete study procedures.

Exclusion criteria included:

▪ Reproductive age;
▪ Systemic and local autoimmune diseases;
▪ Hypothyroidism or hyperthyroidism;
▪ Hormonal and anti-inflammatory therapy [including non-steroidal and steroidal anti-inflammatory drugs, Disease-Modifying Antirheumatic Drugs (DMARDs), or biological therapy];
▪ Cardiac arrhythmias (atrial flutter/fibrillation, atrioventricular conduction disturbances, paroxysmal supraventricular tachycardia);
▪ Acute or chronic myelo- and limfo-prolipherative diseases;
▪ Chronic end-stage kidney, liver or heart failure (eGFR < 15 mL/min/1.75 m^2^; Child Pugh C and D; NT-proBNP > 1000 pg/mL);
▪ Malignant diseases within the last 5 years or their therapy;
▪ Life expectancy less than 6 months;
▪ Mental illness or dementia;
▪ Recent infection (last 4 weeks);
▪ Intra-articular injections within the last 3 months;
▪ Intensive physical therapy within the past 10 days;
▪ Congenital or developmental disorders;
▪ Knee arthroplasty.

Patients not meeting all inclusion criteria or presenting any of the exclusion conditions were not enrolled in the study.

After clinical examination and imaging, patients were referred for laboratory analysis. All patients were informed about the study and required to provide written consent for participation. This study adhered to all applicable guidelines, including the Helsinki Declaration of the World Medical Association (Edinburgh, 2000, UK), to ensure the proper conduct and safety of the participants. During this study, patients did not change their regular therapy or take additional substances or drugs (placebo). The Ethics Committee of the Istrian Health Centers approved this research (Number 2168/01-59-49-01-1/800-20-109) on 22 May 2020.

### 2.2. Clinical Assessment and Measured Parameters

#### 2.2.1. Medical Examinations

The medical examination consisted of medical history review and physical examination. Data from the medical history, including sex, age, smoking status, comorbidities and therapy were obtained using the electronic information system (Softmed2, Vegasoft d.o.o., Ičići, Croatia). The physical examination included palpation and passive movement of the knees, anthropometry (measurement of body height (cm), body weight (kg), waist and hip circumference (cm), waist/hip ratio (WHR), calculation of body mass index (BMI, kg/m^2^), and measurement of arterial blood pressure (mmHg) using an appropriate cuff placed on both arms at baseline, using the Omron M3 Comfort HEM-7134-E (Omron Healthcare Co., Kyoto, Japan).

#### 2.2.2. Radiographic and Ultrasound Imaging

All the patients underwent weight-bearing bilateral anteroposterior radiography of the knee using X-ray DR 400 (Agfa NV, Mortsel, Belgium) to diagnose OA and exclude other potential causes of knee pain. The Kellgren and Lawrence (KL) score was used to detect and grade radiographic disease in OA [24]. The radiographs were evaluated independently by two radiologists, with the final evaluation being a consensus between them. Patients underwent ultrasound using the ultrasonic device Mindray DC-30 (Mindray Bio-Medical Electronics Co., Shenzhen, China) and a linear probe 5–10 MHz (Mindray 7L4P, Shenzhen, China) to comprehensively determine the cause of their knee pain.

### 2.3. Laboratory Analysis

Antecubital venous blood (8–10 mL) was sampled once from each patient in the doctor’s office, and the sample was sent within 2 h to the Central Laboratory of Istrian Health Centre in Pazin for analysis. The erythrocyte sedimentation rate was analysed using Ves-Matic 20 (Diesse, Monteriggioni, Italy). High sensitive C-reactive protein, fasting plasma glucose (FPG), glycosylated haemoglobin (HbA1c), total cholesterol, low-density lipoprotein (LDL), high-density lipoprotein (HDL), triglycerides (Tg), non-HDL cholesterol, uric acid and creatinine were measured using an automatic biochemical analyser (Atellica^®^ Solution CH 930 Analyzer, Siemens Healthineers, Erlangen, Germany) and the atherosclerosis index and estimated glomerular filtration rate (eGFR) were calculated. The remaining serum after the above-mentioned analyses (approximately 500 µL) was collected in a cryo tube (2 mL, Falcon, Lawrence, SAD) and stored at −20 °C until use. Serum oxidised low-density lipoprotein/malondialdehyde (oxLDL/MDA) was measured using ox-LDL/MDA-Adducts kit (cat.no. EIA-5656, DRG Instruments, Marburg, Germany). The absorbance was measured using MRX Revelation microplate reader (Dynex Technologies Inc., Chantilly, VA, USA). In vivo, oxLDL/MDA is created by the action of oxidative stress on apolipoprotein B within the LDL particle, and it denotes the risk for metabolic syndrome and vascular damage [35].

#### Evaluation of Cardiovascular Risk, Atherosclerosis and Renal Function

As part of the overall cardiovascular risk evaluation, we applied the SCORE2/SCORE2-OP algorithms, developed by the European Society of Cardiology [29]. SCORE2 is used for individuals aged 40 to 69 years, whereas SCORE2-OP is designed for those aged 70 years and older. Both models estimate the 10-year risk of first fatal and non-fatal atherosclerotic cardiovascular events based on age, sex, smoking status, systolic blood pressure, and non-HDL cholesterol levels. These tools provide age- and region-specific risk assessments [36].

The atherosclerosis index (AI) was calculated using the following formula:

AI = (Total Cholesterol − HDL-cholesterol)/HDL-cholesterol,

with all lipid parameters expressed in mmol/L [37]. This index reflects the proportion of atherogenic lipoproteins relative to protective high-density lipoproteins and serves as a practical indicator of cardiovascular risk [37].

The estimated glomerular filtration rate (eGFR) was determined using the CKD-EPI 2009 creatinine-based equation, adapted for use with serum creatinine (Scr) in µmol/L, as commonly applied in European clinical laboratories, where the symbol ^ is an exponentiation operator [38]:

For females:

If serum creatinine (Scr) ≤ 62 µmol/L: eGFR = 144 × (Scr/61.9)^–0.329 × (0.993)^Age

If Scr > 62 µmol/L: eGFR = 144 × (Scr/61.9)^–1.209 × (0.993)^Age

For males:

If Scr ≤ 80 µmol/L: eGFR = 141 × (Scr/79.6)^–0.411 × (0.993)^Age

If Scr > 80 µmol/L: eGFR = 141 × (Scr/79.6)^–1.209 × (0.993)^Age.

### 2.4. Assessment of Pain and Functional Capacity

The patients performed a 6 min walk test (6MWT) on a flat, sheltered path near the physicians’ offices, and the length of the walking distance was recorded [39]. Patients were then asked to rate their current level of knee pain on a visual analogue scale (VAS) from 0 to 10. Additionally, physical activity was assessed using the validated International Physical Activity Questionnaire (IPAQ), which estimates activity over the past seven days across walking, moderate-, and vigorous-intensity activity. Activity levels were expressed in Metabolic Equivalent of Task minutes per week (MET-min/week) [40]. MET is a unit that estimates the amount of energy expended during physical activity, where 1 MET represents the energy cost of sitting quietly (approximately 1 kcal/kg/h). The total MET-min/week was calculated by multiplying the duration (minutes per day), frequency (days per week), and the standardised MET value assigned to each activity type: walking (3.3 METs), moderate-intensity activity (4.0 METs) and vigorous-intensity activity (8.0 METs) [40].

### 2.5. Pulse Wave Velocity Measurements

Pulse Wave Velocity (PWV) measurements were performed with a cuff placed on the lower half of the woman’s dominant upper arm using an Agedio^®^ B900 oscillometric device (IEM, Stolberg, Germany) [41]. The device applied Mobil-O-Graph-validated technology for recording brachial blood pressure (BP), including systolic BP, diastolic BP, mean arterial pressure, pulse pressure, and heart rate. Brachial systolic and diastolic BPs were used to calibrate pulse waveforms, which were measured during a 10-s cuff re-inflation. Afterward, the Agedio device reconstructed the aortic pulse waveforms, which, together with aortic characteristic impedance, age, and gender, allowed the estimation of oscillometric PWV. Central systolic blood pressure, central diastolic blood pressure, and the augmentation index were automatically recorded. Measurements were performed at a room temperature of 20–25 °C on working days between 4 and 6 pm.

### 2.6. Statistical Analysis

The normality of the data, statistical analyses, and graphical processing were performed using the Statistica 14.0.0.15 program (TIBCO Software Inc., Palo Alto, CA, USA). Normally distributed continuous variables are compared using the Student’s *t*-test (for two groups) or analysis of variance (ANOVA) for more than two groups and summarised as the arithmetic mean (with 95% confidence intervals) or mean ± standard deviations (SD). Groups with fewer than 50 samples, although following a normal distribution in analysis, were additionally analysed using nonparametric Mann–Whitney U test and presented as the median (range). Categorical data were analysed using Chi-square tests with Yates correction, and the significance was checked with the Fischer’s exact test. Categorical variables were presented as counts and percentages. Pearson correlation coefficients (r) were calculated in the univariate correlation analysis. The correlation was considered strong for a coefficient greater than 0.5, moderate for a coefficient between 0.35 and 0.5, and weak for a coefficient less than 0.35. The level of statistical significance was set at *p* < 0.05.

MedCalc ver. 18.2.1 (MedCalc Software Ltd., Ostend, Belgium) was used to analyse the ability of PWV to distinguish between mild (KL 1-2) and severe (KL 3-4) KOA and to create the optimal statistical cut-off values. A receiver operating characteristic (ROC) curve for PWV and the Youden index were calculated to maximise the sensitivity and specificity of PWV in the univariate model. The area under the ROC curve (AUC) for the score model with a 95% confidence interval (CI) was measured. Sensitivity, specificity, and positive and negative predictive values were calculated for PWV to determine its potential to distinguish mild from severe KOA. The logistic regression method was used to calculate the odds ratio (OR) for PWV, as a predictor of KOA severity (KOA K-L grade 1-2 vs. KOA K-L grade 3-4), first in a univariate model and then in a multivariate model including variables that significantly correlate with KOA severity.

## 3. Results

### 3.1. Recruitment and Allocation of Patients to the Assessment Group

**Figure 1:**
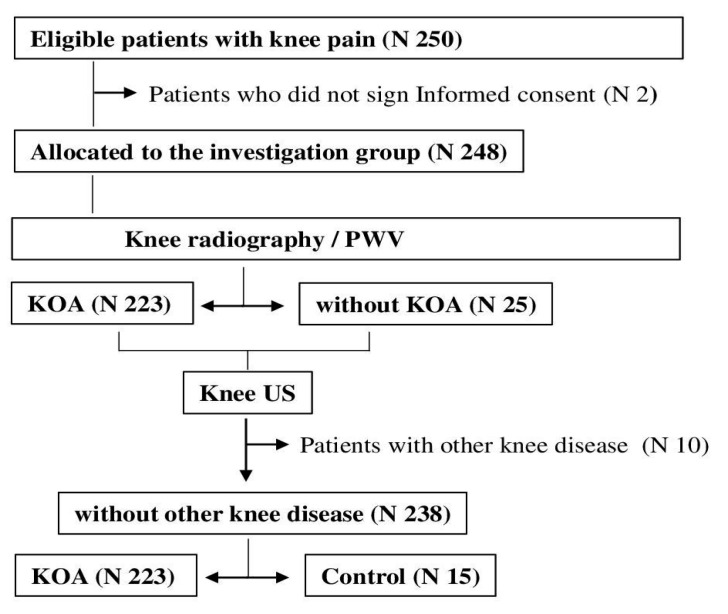
Recruitment and allocation to the assessment group. Abbreviations: KOA—knee osteoarthritis; PWV—pulse wave velocity; US—ultrasound.

The study recruited 250 postmenopausal women who presented to their family doctor with knee pain and who all passed the medical examination (Figure 1). Two patients were excluded from further investigation because they did not sign Informed Consent (Figure 1). The remaining 248 patients had their knees X-rayed. Radiographic KOA was confirmed in 223 patients, according to classification criteria [31], and 25 patients were without KOA. Using ultrasound, we were unable to identify any further joint pathologies in KOA patients (Figure 1). Patients who did not meet radiographic criteria for KOA underwent knee ultrasound to exclude or confirm other joint pathologies. We excluded 10 patients with exacerbation of previously diagnosed spondyloarthritis and included 15 patients with normal knee ultrasound findings as a control group (Figure 1).

#### 3.1.1. Characteristics of Patients with Knee Osteoarthritis

The differences in cardiovascular risk factors between the control group and the group of women with KOA are shown in Table 1.

In the control group, we recruited 15 patients with knee pain without radiological evidence of KOA, who exhibited a normal distribution of the tested parameters. Therefore, we performed a parametric Student’s *t*-test comparison with a larger group of patients with radiological signs of KOA (n 223). However, due to the small number of patients in the control group, we also performed a non-parametric Mann–Whitney U test for comparison with the group of patients with KOA. The results of both tests were similarly significant for BMI, VAS for pain, 6MWT, PWV, and SCORE 2/SCORE 2OP, confirming a higher level of confidence in the results. BMI, VAS for pain, PWV and SCORE 2/SCORE 2OP were increased, while 6MWT was decreased in the patients with KOA, compared with the control group (Table 1). The differences in serum uric acid and eGFR between the groups were of borderline significance (Table 1). Serum uric acid was significantly higher in patients with KOA than in the control using the Mann–Whitney U test (*p* = 0.031), but not when the Student’s *t* -test was used (*p* = 0.091) (Table 1). eGFR was significantly lower in patients with KOA (*p* = 0.008), as shown with Student’s *t*-test only (Table 1). All other parameters tested (age, smoking, systolic and diastolic BP, FPG, HbA1c, total cholesterol, LDL, triglycerides, HDL and non-HDL, atherosclerosis index, oxLDL and creatinine, ESR, and hsCRP) did not differ significantly between the patients with KOA and the control group (Table 1). Comorbidities and medication did not differ between the KOA patients and the control (Table 2).

**Figure 2:**
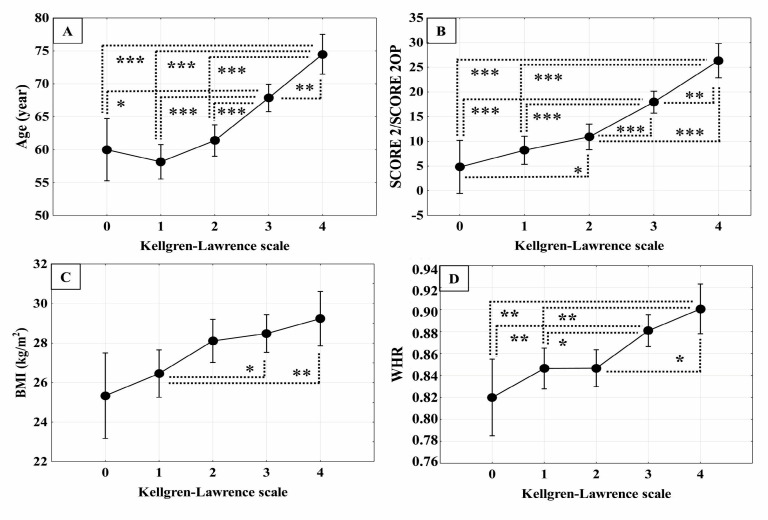
Comparison of classical cardiovascular risk factors in patients with knee osteoarthritis (KOA) of different radiological (Kellgren–Lawrence, KL) grades and controls showing (**A**) age; (**B**) systematic coronary risk assessment (SCORE2 for ages 40–69 years and SCORE2-OP for elderly; (**C**) body mass index (BMI); and (**D**) waist-to-hip ratio (WHR). Number of patients with KL grade 1, n = 46; KL grade 2, n = 57; KL grade 3, n = 74, KL grade 4, n = 31 and controls (KL grade 0), n = 15. Statistical significance: * *p* ≤ 0.05, ** *p* ≤ 0.01, *** *p* ≤ 0.001 (Bonferroni-adjusted *p* values obtained by one-way ANOVA and Tukey’s post hoc test). Data are presented as mean (·) and ±0.95 confidence interval (CI).

Further analysis of classic cardiovascular risk factors among the individual groups of the radiological degree of KOA and the control group is presented in Figure 2.

The ages of patients with KOA of KL grades 3 and 4 were statistically significantly higher than those of patients with KOA grade 2, grade 1 and patients without KOA (grade 0) (Figure 2A). The SCORE 2/SCORE 2OP, which measures total classical cardiovascular risk, was highest in the patients with KOA of KL grade 4 (approximately 25) and showed statistically significantly higher values compared to patients with KOA of grades 3, 2, 1 and 0 (control group) (Figure 2B). Patients with KOA of KL grade 3 showed statistically significantly higher SCORE 2/SCORE 2OP when compared with grades 2, 1 and 0 (Figure 2B). Additionally, SCORE 2/SCORE 2OP was statistically significantly higher in patients with KOA grade 2 compared to the control group (Figure 2B). BMI was statistically significantly higher in patients with KOA grades 3 and 4 compared to patients in grades 1 and 0 (control) (Figure 2C). Additionally, the BMI in patients with KOA grade 2 was statistically significantly higher than in patients with grade 1 (Figure 2C). WHR in patients with KOA grades 4 and 3 was statistically significantly higher than in patients with KOA grade 1 and control group (Figure 2D).

**Figure 3:**
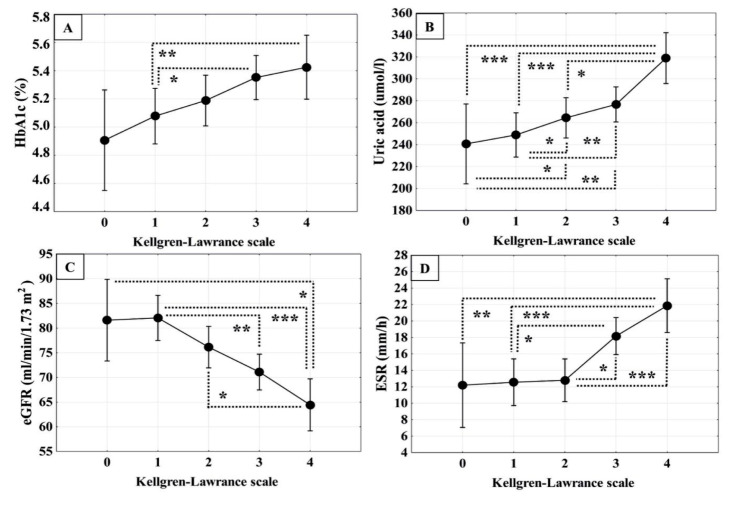
Comparison of non-classical cardiovascular risk factors in patients with knee osteoarthritis (KOA) of different radiological grades and controls Box plots show (**A**) glycated haemoglobin (HbA1c); (**B**) serum uric acid concentration; (**C**) estimated glomerular filtration rate (eGFR); and (**D**) erythrocyte sedimentation rate (ESR). Number of patients with Kellgren–Lawrence (KL) grade 1, n = 46; KL grade 2, n = 57; KL grade 3, n = 74, KL grade 4, n = 31; controls (KL grade 0) n = 15. Statistical significance: * *p* ≤0.05, ** *p* ≤ 0.01, *** *p* ≤ 0.001 (Bonferroni-adjusted *p* values obtained by one-way ANOVA and Tukey’s post hoc test). Data are presented as mean (·) and ± 0.95 confidence interval (CI).

Figure 3 illustrates the differences in non-classical cardiovascular risk factors among the groups with different radiological degrees of KOA and the control group. HbA1c in patients with KOA grade 4 was statistically significantly higher than in patients with KOA grade 1 and the control group (Figure 3A). A statistically significant difference in HbA1c was also found between patients with KOA grades 3 and 1 (Figure 3A). Serum uric acid concentration in patients with KOA grade 4 was statistically significantly higher than in patients belonging to the KL grades 3, 2, 1 and control group (Figure 3B). Additionally, the concentration of uric acid in patients with KOA grade 3 was statistically significantly higher than in the grade 1 group (Figure 3B). The eGFR in patients with KOA of KL grade 4 was statistically significantly lower than in those with KOA grades 2, 1 and the control group (Figure 3C). eGFR in patients with KOA grade 3 was also statistically significantly lower than in the grade 1 group (Figure 3C). The ESR in patients with KOA of KL grade 4 was statistically significantly higher than in those with KOA of KL grade 1 and 0 (control) (Figure 3D). Furthermore, ESR in patients with KOA grade 3 was statistically significantly higher than in the grade 2 and grade 1 groups (Figure 3D).

#### 3.1.2. Correlation of Cardiovascular Risk Factors with Radiological Grade of Knee Osteoarthritis

**Figure 4:**
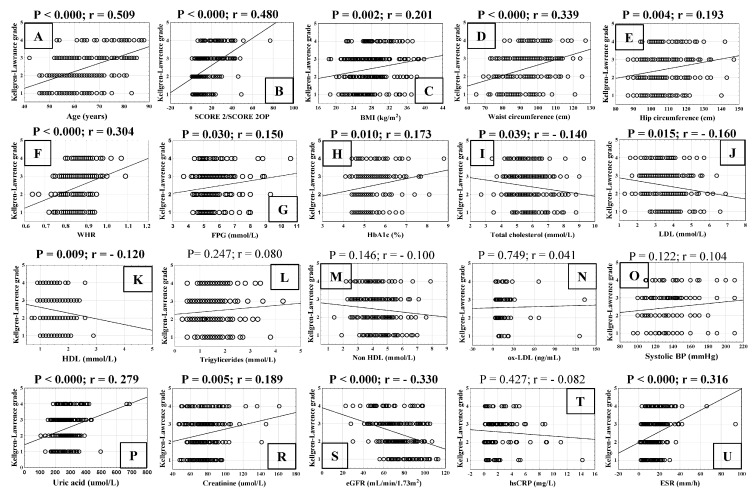
Correlation of Kellgren–Lawrence grade (1–4) of knee osteoarthritis (KOA) and cardiovascular risk factors Linear graphs show (**A**) age; (**B**) systematic coronary risk assessment (SCORE2 for ages 40–69 years and SCORE2-OP for elderly; (**C**) body mass index (BMI); (**D**) waist circumference; (**E**) hip circumference; (**F**) waist-to-hip ratio (WHR); (**G**) fasting plasma glucose (FPG); (**H**) glycated haemoglobin (HbA1c); (**I**) total cholesterol; (**J**) low-density lipoprotein (LDL); (**K**) high-density lipoprotein (HDL); (**L**) triglycerides; (**M**) non—HDL; (**N**) oxidised low-density lipoprotein (ox-LDL); (**O**) systolic blood pressure (BP); (**P**) serum uric acid concentration; (**R**) creatinine; (**S**) estimated glomerular filtration rate (eGFR); (**T**) high sensitive C-reactive protein (hsCRP) and (**U**) erythrocyte sedimentation rate (ESR). Statistical significance (*p*) and correlation coefficient (r) are shown above dot plots.

We analysed the correlation between cardiovascular risk factors and the radiological grade of KOA in all patients (Figure 4). The KOA grade (KL scale) correlated positively with age and SCORE 2/SCORE 2OP (Figure 4A,B), BMI (Figure 4C), waist circumference (Figure 4D), hip circumference (Figure 4E), WHR ratio (Figure 4F), FPG (Figure 4G), and HbA1c (Figure 4H). The concentration of total cholesterol (Figure 4I), LDL (Figure 4J) and HDL (Figure 4K) statistically significantly negatively correlated with KOA grade, whereas KOA grade was not related to TG (Figure 4L), non-HDL (Figure 4M), oxLDL (Figure 4N), and systolic BP (Figure 4O). The serum concentration of uric acid (Figure 4P) and creatinine (Figure 4R) statistically significantly positively correlated with KOA radiologic grade, whereas eGFR showed the opposite trend, negatively correlating with KOA grade (Figure 4S). The inflammatory parameter hsCRP (Figure 4T) did not correlate with the KOA grade, but ESR showed a statistically significant positive correlation with the KOA grade (Figure 4U).

#### 3.1.3. Correlation of Cardiovascular Risk Factors with Pulse Wave Velocity

**Figure 5:**
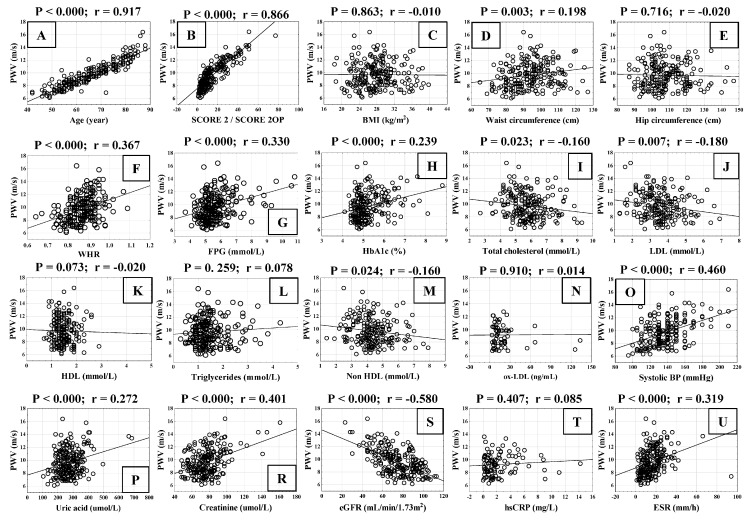
Correlation of pulse wave velocity (PWV) and cardiovascular risk factors in patients with knee osteoarthritis (KOA) Linear graphs show (**A**) age; (**B**) systematic coronary risk assessment (SCORE2 for ages 40–69 years and SCORE2-OP for elderly); (**C**) body mass index (BMI); (**D**) waist circumference; (**E**) hip circumference; (**F**) waist-to-hip ratio (WHR); (**G**) fasting plasma glucose (FPG); (**H**) glycated haemoglobin (HbA1c); (**I**) total cholesterol; (**J**) low-density lipoprotein (LDL); (K) high-density lipoprotein (HDL); (**L**) triglycerides; (**M**) non-HDL; (**N**) oxidised low-density lipoprotein (ox-LDL); (**O**) systolic blood pressure (BP); (**P**) serum uric acid concentration; (**R**) creatinine; (**S**) estimated glomerular filtration rate (eGFR); (**T**) high sensitive C-reactive protein (hsCRP); and (**U**) erythrocyte sedimentation rate (ESR). Statistical significance (*p*) and correlation coefficient (r) are shown above dot plots.

The interrelationship between PWV and cardiovascular risk factors in all patients with KOA is shown in Figure 5. PWV showed a statistically significant positive correlation with age and SCORE 2/SCORE 2OP (Figure 5A,B). BMI was not correlated with PWV (Figure 5C). Waist circumference was significantly positively correlated with PWV (Figure 5D), but hip circumference was not (Figure 5E), which resulted in WHR being statistically significantly positively correlated with PWV (Figure 5F). FPG (Figure 5G) and HbA1c (Figure 5H) showed statistically significant positive correlation with PWV. Total cholesterol (Figure 5I) and LDL (Figure 5J) statistically significantly negatively correlated with PWV, whereas HDL and triglycerides were not correlated with PWV (Figure 5K,L). Non-HDL cholesterol significantly negatively correlated with PWV (Figure 5M), and oxLDL was not correlated with PWV (Figure 5N). Systolic BP (Figure 5O), serum uric acid concentration (Figure 5P), and creatinine (Figure 5R) statistically significantly positively correlated with PWV, but eGFR showed a statistically significant negative correlation (Figure 5S). hsCRP did not correlate with PWV (Figure 5T), whereas ESR significantly positively correlated with PWV (Figure 5U).

#### 3.1.4. Correlation of Functional Capacity of Patients with Radiological Grade of Knee Osteoarthritis and Pulse Wave Velocity

**Figure 6:**
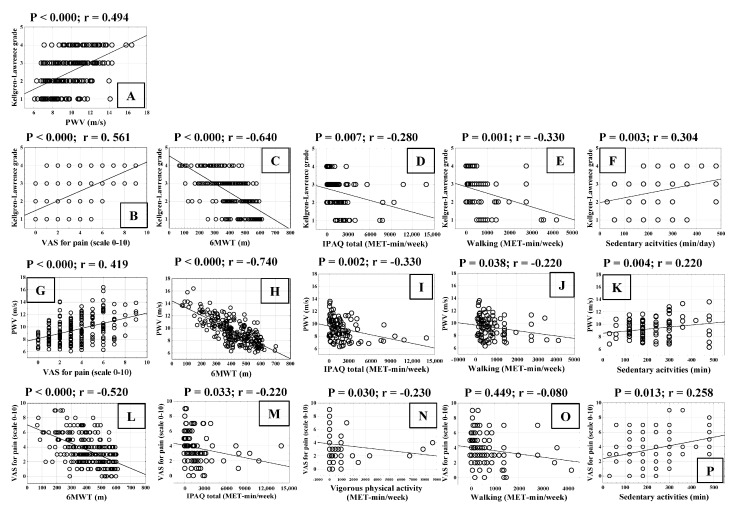
Correlation of Kellgren–Lawrence grade (1–4) (**A**–**F**), pulse wave velocity (PWV) (**G**–**K**) and VAS for pain (**L**–**P**) with functional capacity parameters in patients with knee osteoarthritis (KOA). Abbreviations: IPAQ—The International Physical Activity Questionnaire; 6MWT—6 min walk test. Statistical significance (*p*) and correlation coefficient (r) are shown above dot plots.

We analysed the relationship between the radiological grade of KOA and PWV, as well as the possible association of these parameters with pain intensity and functional ability in patients with KOA (Figure 6). Radiological KL grade correlated statistically significantly positively with PWV and VAS for pain (Figure 6A,B). The radiological grade of KOA showed a statistically significant negative correlation with the 6MWT (Figure 6C), IPAQ-total (Figure 6D), and walking (Figure 6E), whereas it exhibited a statistically significant positive correlation with sedentary activities (Figure 6F). Similarly, PWV was statistically significantly positively correlated with VAS for pain (Figure 6G) and negatively correlated with 6MWT (Figure 6H), IPAQ-total (Figure 6I), and walking (Figure 6J). PWV also showed a statistically significant positive correlation with sedentary activities (Figure 6K). VAS for pain showed a statistically significant negative correlation with 6MWT (Figure 6L), IPAQ-total (Figure 6M), and vigorous physical activity (Figure 6N). Walking was not associated with VAS for pain (Figure 6O), but VAS for pain correlated statistically significantly positively with sedentary activities (Figure 6P).

#### 3.1.5. Comparison of Cardiovascular Risk Assessment Between Mild and Severe Knee Osteoarthritis Groups

The KOA 1–2 groups included 108, and the KOA 3–4 groups included 115 patients (Table 3). PWV, age, SCORE 2/SCORE 2OP, VAS for pain, WHR, waist circumference, hip circumference, BMI, cigarette smoking, FPG, HbA1c, uric acid, creatinine, ESR, hsCRP and sedentary activity were all statistically significantly higher in the groups of KOA KL grade 3–4 when compared with KOA KL 1–2 groups, as calculated with Student’s *t*-test and Mann–Whitney U test (Table 3). There were no significant differences in systolic BP and metabolic parameters such as total cholesterol, triglycerides, HDL, non-HDL, atherosclerosis index and oxLDL between the groups (Table 3). Women with KOA of KL grades 3–4 had statistically lower diastolic BP, eGFR, LDL, and 6MWT, and they walked less than the women with KOA of KL grades 1–2 in both tests applied (Table 3). Physical activity expressed as IPAQ total, vigorous, and moderate, was significantly lower in the KOA 3–4 groups compared to the KL 1–2 groups, as determined by the Mann–Whitney U test, but not by the Student’s *t*-test (Table 3). The results in the mentioned categories showed a normal distribution; however, not all participants were willing to complete the IPAQ questionnaire.

#### 3.1.6. Difference in Pulse Wave Velocity Between Mild and Severe Forms of Knee Osteoarthritis

**Figure 7:**
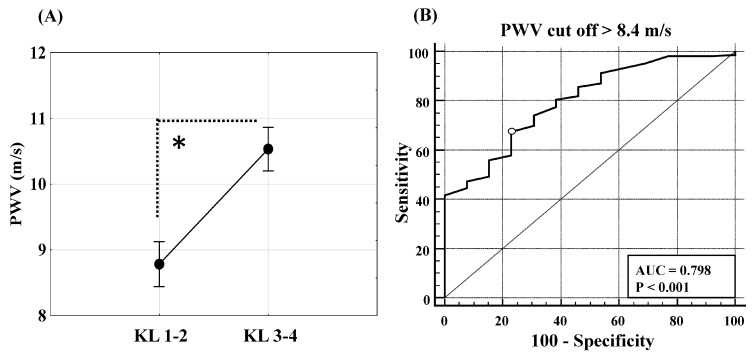
Difference in pulse wave velocity (PWV) during knee osteoarthritis (KOA) progression. Pulse wave velocity (PWV) between patients with mild form (Kellgren–Lawrence grades 1-2, n 115) and severe form (Kellgren–Lawrence grades 3-4, n 108) of KOA (**A**), and the area under the receiver operating characteristics curve (AUC) for PWV at the optimal cut-off value of > 8.4 m/s to distinguish severe form of KOA with respect to patients with mild form of KOA. Level of statistical significance is calculated using (**A**) the Mann–Whitney U test * *p* = 0.001 and (**B**) the receiver operating characteristic analysis as indicated in the plot.

Patients with mild KOA (KL grades 1-2) showed statistically significantly lower PWV [8.4 m/s (6.1–7.1), mean (95% CI)], compared to patients with severe KOA (KL grades 3-4) [10.5 m/s (6.8–16.4)], as diagnosed by radiography (Figure 7A, *p* = 0.001). This led us to perform a ROC curve analysis, which indicated that PWV > 8.4 m/s was the cut-off value for distinguishing mild from severe KOA (Figure 7B). The AUC for PWV at the optimal cut-off value of > 8.4 m/s was 0.798 (Figure 7B), with a 95% CI ranging from 0.740 to 0.849. PWV can distinguish mild from severe KOA, with a specificity of 76.9% and sensitivity of 67.5%. The positive predictive value of PWV was 97.9% (0.95 CI, 94.6% to 99.2%), and the negative predictive value was 12.9% (95% CI, 9.5% to 17.6%).

A patient with PWV > 8.4 m/s has a 1.77-fold higher chance of developing severe KOA than a patient with a lower PWV, according to a univariate model of the logistic regression method, similar to age, BMI, and waist circumference (OR 1.1). However, PWV in a multivariate model including variables that significantly correlated with KOA severity (age, BMI, SCORE 2/SCORE 2OP) was not an independent prognostic parameter for KOA progression.

Based on the cut-off value for PWV, patients were allocated into dichotomous groups: the group with PWV ≤ 8.4 m/s (n 72) and the group with PWV > 8.4 m/s (n 151), and their characteristics were compared using Student’s *t*-test and shown in Table 4.

PWV was 10.69 ± 1.61 (mean ± SD) in the group with PWV > 8.4 m/s and was statistically significantly higher (*p* < 0.001) than in the group with PWV ≤ 8.4 m/s (7.57 ± 0.60) (Table 4). Radiological grade, age, SCORE 2/SCORE 2OP, and VAS for pain were all statistically significantly higher in the group with PWV > 8.4 m/s (Table 4). WHR and waist circumference were also significantly higher in the group with PWV > 8.4 m/s, while similar values of hip circumference and BMI were found in both groups applying Student’s *t*-test and Mann–Whitney U test (Table 4). The duration of cigarette smoking and serum concentration of total cholesterol were lower in the group with PWV > 8.4 m/s than in the group with PWV ≤ 8.4 m/s, as calculated using the Mann–Whitney U test, but not Student’s *t*-test. The diastolic BP did not differ significantly between the dichotomous groups, while systolic BP, FPG, HbA1c, serum uric acid and creatinine concentrations were all statistically significantly higher in the group with PWV > 8.4 m/s using both tests (Table 4). The eGFR was statistically significantly lower in the group with higher PWV. ESR was statistically higher in the group with PWV > 8.4 m/s, but hsCRP did not significantly change, nor did levels of LDL, triglycerides, HDL and non-HDL, atherosclerosis index and concentration of oxLDL, as calculated with Student’s *t*-test and the Mann–Whitney U test (Table 4). The 6MWT was statistically significantly lower in the group with the PWV > 8.4 m/s in both tests used. Total and vigorous physical activity determined using IPAQ decreased in the group with PWV > 8.4 m/s, as calculated by Student’s *t*-test, but not by the Mann–Whitney U test. The moderate physical activity, walking, and sedentary activity groups did not differ significantly between the dichotomous groups (Table 4).

## 4. Discussion

This is, to our knowledge, the first study to show that the estimated oscillometric PWV value, an essential tool for assessing arterial stiffness from reconstructed aortic pulse waveform [41], is statistically significantly different between mild and severe KOA at a PWV cut-off value > 8.4 m/s, with significant specificity (76.92%) and sensitivity (67.46%). The estimated ocillometric PWV increased with the radiographic degree of KOA and provided a fast assessment of KOA progression without additional radiation in postmenopausal women, offering a quick, cost-effective, and practical exam in a doctor’s office. KOA affects more women than men and progresses rapidly, especially after menopause, when women have an increased risk for arterial calcifications and cardiovascular diseases [25]. Estrogen has been shown to have widely recognised cardioprotective benefits, including improving endothelial function, increasing nitric oxide bioavailability, and decreasing arterial stiffness, through anti-inflammatory and vasodilatory processes [42]. To reduce hormonal variability and minimise the confounding effects of estrogen on arterial stiffness outcomes, we recruited postmenopausal women, whose levels of progesterone and estrogen decline [43]. By focusing on this group, we were able to produce a more uniform sample and ensure our findings are particularly relevant to a population that is most impacted by a combination of these factors. Additionally, the estimated ocillometric PWV value was significantly higher in men than in women, although the difference gradually diminished with age [41]. Recently, aortic stiffness, measured by carotid–femoral PWV, was shown to be independently associated with KOA grade in a mixed group of men and women after adjusting for the classical cardiovascular risk factors and hsCRP, whereas the correlation with femoral-ankle PWV was borderline. The authors [41] used a novel sophisticated ultrasound technique, which is not widely available due to its high cost and the need for highly trained experts [26]. The stiffening of the aorta seems to be associated with knee OA rather than calcification of the lower limb arteries, as only carotid-femoral PWV was increased in patients with OA compared to healthy controls [26]. The relationship between OA and arterial stiffness is currently being investigated in the context of lifestyle-related diseases and classical cardiovascular risk factors [44]. In line with these efforts, we found that statistically significant increases in age, SCORE 2/SCORE 2OP, VAS for pain, WHR, waist circumference, FPG, HbA1c, uric acid, creatinine, and ESR occurred together with higher PWV in the severe KOA group (KL 3-4 grade), as well as in the dichotomous group with PWV > 8.4 m/s compared to mild KOA (KL 1-2 grade) or the dichotomous group with PWV ≤ 8.4 m/s. It demonstrates that the value of PWV estimated using an oscillometric device, although it is not an independent predictor for KOA progression, can indicate the aforementioned cardiometabolic risk factors in postmenopausal women with KOA, which is further supported by the lower eGFR and 6MWT in the severe KOA group and the group with PWV > 8.4 m/s. The systolic pressure was higher and the IPAQ total and vigorous physical activity were borderline discounted in the group with PWV > 8.4 m/s but did not differ significantly or unambiguously between mild and severe KOA; therefore, PWV may not reflect their values during KOA progression.

In accordance, the aortic stress and distensibility values were significantly lower, while the inflammatory marker C-reactive protein was higher in the Kellgren–Lawrence OA grade 4 group compared to the other groups [45], as were ESR and parameters related to metabolic inflammation (HbA1c, uric acid, WHR, SCORE 2/SCORE 2OP, and age), which are presented for the first time in this manuscript. This indicates the essential role of OA in aortic stiffening, although the aorta naturally stiffens with aging and predicts cardiovascular morbidity and all-cause mortality, as advanced age is associated with high cardiovascular risk factors [46]. An increase in plasma LDL particles is considered to be associated with atherogenesis, a prototype of low-grade inflammation [47], as well as OA [15]. However, peripheral blood concentrations of HDL, LDL, and total cholesterol did not correlate with OA grade or PWV in our study. In accordance, no slowing of structural progression was observed during treatment of hypercholesterolemia [48]. This supports the idea that LDL particles themselves do not cause arteriosclerosis or promote OA [48]. Only products of oxLDL, which increases significantly in inflammatory conditions, might be harmful to blood vessels and joints [49,50]. In vitro, oxLDL did not affect the viability of human chondrocytes and decreased their pro-inflammatory cytokine production, thereby protecting the joint [48], and serum oxLDL did not correlate with PWV and KOA grade in vivo. In the synovial fluid of patients with OA, oxLDL has been demonstrated, and the concentration of its soluble receptor LOX-1 positively correlates with the radiological grade of OA [50].

Approximately 90% of patients with OA suffer from arterial hypertension, which increases artery wall stiffness [18] and the risk for coronary artery disease [51]. Mean blood pressure increases with increasing carotid-femoral and femoral-ankle PWV [26], as does systolic blood pressure, which also increases along with high estimated oscillometric PWV values. More than 30% of OA patients have type II diabetes [52] and an abdominal obesity phenotype, both of which increase arterial stiffness when compared with healthy controls [53]. Glycotoxicity represents a risk factor for KOA [54], causing cartilage degradation and abnormal remodeling of subchondral bone in obese patients [55]. The majority of women in the study were overweight. BMI and hip circumference statistically significantly positively correlated with OA grade, but not with PWV, indicating a stronger association between body mass (i.e., mechanical load) and KOA [6], when compared with PWV. Obesity, characterised by increased waist circumference, WHR, FPG, HbA1c, age, and the SCORE 2/SCORE 2OP, statistically significantly positively correlated with KOA grade and the PWV, highlighting the importance of metabolic parameters in postmenopausal women with KOA. On the other hand, conditions characterised by varying degrees of inflammation increase arterial stiffness [25], such as OA [15], diabetes [53], and chronic kidney disease [56]. This aligns with the positive correlation of ESR with PWV and OA grade in this investigation. Serum uric acid concentration acts proinflammatory and is positively correlated with arterial stiffness [57], carotid-femoral PWV [21], and OA grade in this study. Uric acide likely promotes various cartilage damages [58], although it cannot induce OA by itself [59]. In the context of inflammation, calcifications in the intima and tunica media represent significant complications of chronic kidney disease [56], increasing vascular resistance [60]. Endothelial dysfunction, arterial hypertension, and diabetes, although major cause of chronic kidney disease [61], cannot explain entirely the development of arterial calcification in these patients, which is attributed to the mineral bone metabolism and inflammation [62]. The prevalence of vascular calcifications tends to increase as the glomerular filtration rate declines in patients with chronic kidney disease [63]. This is consistent with the results showing that creatinine correlates positively, and eGFR negatively, with PWV and KOA grade in postmenopausal women. Additionally, eGFR was significantly reduced in OA grades 3 and 4 compared to grade 1 group [56], and in the control group without KOA according to our results, reflecting increased inflammation in chronic kidney disease. Hyperglycemia affects the nociceptive pathway, intensifying pain [64]. In patients with KOA, joint pain positively correlated with the radiographic grade of OA and with PWV, suggesting that chronic pain could be a clinical sign of the arterial stiffness [30]. Increased knee pain decreases functional capacity of patients, such as in 6MWT, IPAQ total, and the ability to perform strenuous physical activities, which likely exacerbates classical cardiovascular risk factors, supporting a sedentary lifestyle. In the group with PWV > 8.4 m/s, 6MWT was significantly reduced, whereas IPAQ total and vigorous physical activity were marginally decreased, indicating a lack of beneficial effect of physical activity on endothelial dysfunction and vascular health. This is consistent with the fact that 6MWT was negatively associated with arterial stiffness and PWV in our investigation, in accordance with the findings of other research groups [65].

There are several weaknesses of the study. This is a cross-sectional study, so causal relationships cannot be established. Another limitation was the difficulty in recruiting a significant number of postmenopausal women in the control group. The study recruited only postmenopausal women, which limits generalisability to other subpopulations. Hormonal and metabolic differences between sexes and age groups may influence both OA progression and cardiovascular risk profiles.

## 5. Conclusions

PWV increases in postmenopausal women with KOA progression within the context of inflammation supported by metabolic factors and represents an easily interpretable parameter for assessing the overall risk for cardiovascular diseases, obtained by a simple, non-invasive, and time-efficient oscillometric method that is applicable in a real-life primary care setting. Future studies with larger and more balanced control groups are warranted to further validate our findings and improve the generalisability of the results.

## Figures and Tables

**Table 1:** Characteristics of patients with knee osteoarthritis (KOA) and controls.

	Control (N 15)		Patients with KOA (N 223)		Man-Whitney U Test *p* Value	Student’s *t* Test *p* Value
	Median (Range)	Mean ± SD	Median (Range)	Mean ± SD		
Kellgren-Lawrence grade	-	-	3 (1–4)			
Age (years)	61 (50–72)	60 ± 7	65 (42–96)	65 ± 11	0.073	0.070
BMI (kg/m^2^)	25 (20–32)	25.60 ± 3.10	28 (18–45)	28 ± 4.40	0.012	0.010
VAS for pain (scale 0–10)	1 (0–5)	1.60 ± 2	3 (0–9)	3.5 ± 2	0.010	<0.001
6MWT (m)	588 (356–702)	575 ± 94	401 (68–612)	406 ± 126	<0.001	<0.001
PWV (m/s)	7.30 (6.40–10.90)	7.70 ± 1.30	9.40 (6.10–16.40)	9.70 ± 2	<0.001	<0.001
SCORE 2/SCORE 2OP	4 (1–14)	4.80 ± 3.80	11 (1–77)	15.70 ± 12.70	<0.001	0.002
Cigarette smoking (years)	0 (0–45)	4.10 ± 12	0 (0–46.5)	6 ± 11	0.638	-
Systolic BP (mmHg)	120 (103–160)	127 ± 20	138 (95–210)	138 ± 21	0.069	0.063
Diastolic BP (mmHg)	80 (64–90)	79 ± 9.50	80 (55–110)	80 ± 11.40	0.810	0.864
FPG (mmol/L)	5.50 (4.50–6.50)	5.40 ± 0.62	5.40 (3.70–12)	5.70 ± 1	0.300	0.180
HbA1c (%)	4.95 (4.40–5.40)	4.93 ± 0.29	5 (4.10–8.80)	5.23 ± 0.72	0.104	0.062
Total cholesterol (mmol/L)	5 (4.60–8.30)	6 ± 1.20	5.70 (2.70–9.30)	5.80 ± 1.10	0.422	0.295
LDL (mmol/L)	3.70 (2.60–6.50)	3.90 ± 1.10	3.50 (1.30–6.90)	3.60 ± 0.99	0.264	0.174
Triglycerides (mmol/L)	1 (0.60–1.90)	1.10 ± 0.40	1.30 (0.30–4.30)	1.45 ± 0.65	0.060	0.069
HDL (mmol/L)	1.70 (7–2)	1.60 ± 0.30	1.40 (0.70–2.90)	1.5 ± 0.36	0.160	0.325
Non-HDL (mmol/L)	4.30 (2.90–2.70)	4.50 ± 1.30	4.20 (1.40–7.90)	4.30 ± 1	0.640	0.467
Atherosclerosis index	2.06 (1.40–5)	2.43 ± 0.99	2.40 (0.90–5.75)	2.50 ± 0.82	0.991	0.748
oxLDL (pg/mL)	12.80 (7.20–162.50)	48.80 ± 75.90	13 (3.40–202.80)	24.20 ± 36.30	0.800	0.223
Uric acid (μmol/L)	225 (178–401)	240 ± 58	261 (105–685)	271 ± 77	0.031	0.091
Creatinine (μmol/L)	77.50 (61–88)	76 ± 7.20	75 (47–161)	76.70 ± 16.80	0.090	0.781
eGFR (mL/min/1.73 m^2^)	79 (63–101)	80.60 ± 11.70	73 (23–113)	73.80 ± 17.50	0.070	0.008
ESR (mm/h)	12 (2–37)	12.60 ± 9.50	14 (1–94)	16 ± 11	0.080	0.160
hsCRP (mg/L)	1.30 (0.60–2.60)	1.34 ± 0.80	1.45 (0–14.20)	2.37 ± 2.50	0.460	0.326

Abbreviations: BMI—body mass index; BP—blood pressure; eGFR—estimated glomerular filtration rate; ESR—erythrocyte sedimentation rate; FPG—fasting blood glucose; HbA1c—glycated haemoglobin; HDL—high-density lipoprotein; hsCRP—high-sensitivity C-reactive protein; LDL—low-density lipoprotein; oxLDL—oxidative modification of low-density lipoprotein; PWV—pulse wave velocity; SCORE 2/SCORE 2OP—systematic coronary risk evaluation, age 40–69 years/SCORE2-OP older persons, age 70–89 years; SD—standard deviation; 6MWT—6 min walk test; VAS—visual analog scale. Statistical significance (*p*) was calculated using Mann–Whitney U test and Student’s *t*-test, with *p* ≤ 0.05 considered significant.

**Table 2:** Comorbidities and medication in patients with knee osteoarthritis (KOA) and controls.

Comorbidities and Medication	Control (N 15) N (%)	KOA (N 223) N (%)	Chi-Squere Test	Yates’s Correction	Fisher’s Exact Test
Cardiovascular diseases	5 (33.33)	112 (50.22)	0.205	0.206	0.158
Diabetes mellitus	3 (20.00)	79 (35.42)	0.223	0.349	0.175
Chronic respiratory diseases	4 (26.66)	53 (23.76)	0.548	0.787	0.373
Gastrointestinal disorders	6 (40.00)	126 (56.50)	0.213	0.328	0.164
Neurological diseases	2 (13.33)	43 (19.28)	0.569	0.818	0.434
ACE inhibitors	3 (20.00)	74 (33.18)	0.290	0.440	0.224
ARB	1 (6.66)	15 (6.72)	0.992	0.600	0.733
Beta-blockers	4 (26.66)	82 (36.77)	0.430	0.609	0.311
Calcium channel blocker	1 (3.66)	17 (7.62)	0.892	0.712	0.684
Diuretics	1 (3.66)	11 (4.93)	0.766	0.754	0.551
Statins	3 (20.00)	76 (34.08)	0.262	0.402	0.204
Metformin	3 (20.00)	77 (34.52)	0.235	0.365	0.184
Dipeptidyl Peptidase IV	1 (6.66)	46 (20.62)	0.188	0.327	0.164
Allopurinol	2 (13.33)	28 (12.55)	0.930	0.753	0.588
Proton pump inhibitors	6 (40.00)	104 (46.63)	0.617	0.816	0.411
Paracetamol	5 (33.33)	120 (53.81)	0.545	0.739	0.375
Tramadol	2 (13.33)	49 (21.97)	0.429	0.642	0.338

Abbreviations: ACE—angiotensin-converting enzyme; ARB—angiotensin II receptor blocker. Categorical data were analysed using Chi-square test with Yates’s correction and Fisher’s exact test; results are presented as counts (N) and percentages (%).

**Table 3:** Characteristics of patients with mild knee osteoarthritis (KOA) of Kellgren–Lawrence grades 1–2 and severe KOA of Kellgren–Lawrence grades 3–4.

	KOA 1–2 (N 108)		KOA 3–4 (N 115)		*p* Value	
	Median (Range)	Mean SD	Median (Range)	Mean SD	Mann-Whitney U Test	Student’s *t* Test
PWV (m/s)	8.50 (6.10–14.10)	8.40 ± 1.63	10.40 (6.80–16.40)	10.50 ± 1.96	<0.001	<0.001
Age (years)	58 (46–83)	59.96 ± 8.76	71 (42–88)	69.99 ± 10.50	<0.001	<0.001
SCORE 2/SCORE 2OP	7.25 (1–49)	7.00 ± 6.65	19 (1–77)	20.92 ± 13.78	<0.001	<0.001
VAS for pain (scale 0–10)	2 (0–6)	2 ± 1.44	5 (0–9)	4.70 ± 2.03	<0.001	<0.001
WHR	0.86 (0.64–0.95)	0.84 ± 0.06	0.89 (0.74–1.09)	0.89 ± 0.06	<0.001	<0.001
Waist circumference (cm)	89 (65–125)	90.25 ± 11.28	97 (73–127)	97.40 ± 11.01	<0.001	<0.001
Hip circumference (cm)	104 (87–143)	105.70 ± 11	108 (87–147)	110.30 ± 10.61	0.005	0.002
BMI (kg/m^2^)	27.10 (18.70–38.20)	27.36 ± 4.14	28.20 (18.40–44.80)	28.73 ± 4.52	0.013	0.002
Cigarette smoking (years)	0 (0–46.50)	0.72 ± 13.31	0 (0–40)	2.29 ± 7.10	0.002	<0.001
Diastolic BP (mmHg)	80 (60–110)	88.80 ± 11.60	80 (55–105)	77.77 ± 10.61	0.039	0.021
Systolic BP (mmHg)	130 (95–210)	136 ± 20.50	140 (100–210)	139 ± 21.17	0.360	0.388
FPG (mmol/L)	5.40 (4.30–9.30)	5.53 ± 0.86	5.60 (3.70–12.10)	5.87 ± 1.22	0.019	0.021
HbA1c (%)	4.90 (4.40–8.10)	5.13 ± 0.64	5.20 (4.10–8.80)	5.37 ± 0.77	0.009	0.014
Uric acid (µmol/L)	254 (105–496)	257.30 ± 63	275 (152–685)	290.30 ± 82.52	0.003	0.001
Creatinine (µmol/L)	73 (48–141)	74.68 ± 13.55	78 (47–161)	79.17 ± 18.75	0.046	0.043
eGFR (mL/min/1.73 m^2^)	79 (30–113)	78.85 ± 14.85	67 (23–105)	69 ± 18.26	<0.001	<0.001
ESR (mm/h)	12 (1–36)	12.68 ± 7.23	18 (2–94)	19.35 ± 12.32	<0.001	<0.001
hsCRP (mg/L)	1.25 (0–24.10)	2.45 ± 3.41	2.30 (0.10–6.40)	4 ± 7.23	0.001	0.035
Total cholesterol (mmol/L)	5.80 (2.70–8.80)	5.90 ± 1.13	5.60 (3.40–9.30)	5.65 ± 1.07	0.075	0.972
LDL (mmol/L)	3.60 (1.30–6.90)	3.71 ± 1.01	3.40 (1.60–6.40)	3.42 ± 0.95	0.022	0.028
Triglycerides (mmol/L)	1.30 (0.30–3.80)	1.40 ± 0.65	1.30 (0.50–4.30)	1.49 ± 0.65	0.195	0.333
HDL (mmol/L)	1.50 (0.70–2.90)	1.52 ± 0.38	1.40 (0.80–2.60)	1.45 ± 0.33	0.098	0.143
Non-HDL (mmol/L)	4.25 (1.40–7.60)	4.38 ± 1.08	4.1 (2.20–7.90)	4.56 ± 3.77	0.242	0.642
Atherosclerosis index	2.45 (1–5.75)	2.54 ± 0.82	2.40 (0.90–4.90)	2.47 ± 0.83	0.529	0.505
oxLDL (pg/mL)	13.80 (3.35–154.55)	25.63 ± 34.94	11.60 (3.90–202.80)	23.06 ± 37.78	0.605	0.779
6MWT (m)	501 (125–612)	477 ± 102	351 (68–576)	337 ± 110	<0.001	<0.001
IPAQ total (MET-min/week)	1890 (0–9373)	2366 ± 2212	643.50 (0–13878)	1430 ± 2450	<0.001	0.063
Vigorous physical activity (MET-min/week)	80 (0–7840)	564 ± 1334	0 (0–8640)	422 ± 1540	0.009	0.646
Moderate physical activity (MET-min/week)	720 (0–5040)	829 ± 909	120 (0–5040)	460 ± 958	<0.001	0.065
Walking (MET-min/week)	594 (0–4158)	973 ± 1007	313.50 (0–2722)	578 ± 666	0.014	0.026
Sedentary activity (min/day)	180 (30–480)	204 ± 101	240 (60–480)	251 ± 112	0.036	0.039

Abbreviations: BMI—body mass index; BP—blood pressure; eGFR—estimated glomerular filtration rate; ESR—erythrocyte sedimentation rate; FPG—fasting blood glucose; HbA1c—glycated haemoglobin; HDL—high-density lipoprotein; hsCRP—high-sensitivity C-reactive protein; IPAQ—International Physical Activity Questionnaire; LDL—low-density lipoprotein; MET—metabolic equivalent of task; oxLDL—oxidative modification of low-density lipoprotein; PWV—pulse wave velocity; SCORE 2/SCORE 2OP—systematic coronary risk evaluation, age 40–69 years/SCORE2-OP older persons, age 70–89 years; SD—standard deviation; 6MWT—6 min walk test; VAS—visual analog scale; WHR—waist-hip ratio. Statistical significance (*p*) was calculated with Student’s *t*-test and Mann–Whitney U test with *p* ≤ 0.05 considered significant.

**Table 4:** Differences between dichotomous groups of patients with knee osteoarthritis (KOA) based on PWV cut-off value > 8.4 m/s.

	PWV ≤ 8.4 m/s (N 72)		PWV > 8.4 m/s (N 151)		*p* Value	
	Median (Range)	Mean ± SD	Median (Range)	Mean ± SD	Mann- Whitney U Test	Student’s *t* Test
PWV (m/s)	7.70 (6.10–8.40)	7.57 ± 0.60	10.50 (8.50 -14.30)	10.69 ± 1.61	<0.001	<0.001
Kellgren-Lawrence grade	2 (1–4)	1.86 ± 0.87	3 (1–4)	2.74 ± 0.94	<0.001	<0.001
Age (years)	54 (42–64)	54.09 ± 5.02	70 (50–88)	70.37 ± 8.84	<0.001	<0.001
SCORE 2/SCORE 2OP	5.50 (1–14)	6.13 ± 3.32	17 (2.50–51)	20.28 ± 13	<0.001	<0.001
VAS for pain (scale 0-10)	2 (0–7)	2.90 ± 1.87	4 (0–9)	3.93 ± 2.12	<0.001	<0.001
WHR	0.83 (0.73–0.98)	0.84 ± 0.06	0.89 (0.64–1.09)	0.88 ± 0.06	<0.001	<0.001
Waist circumference (cm)	87 (69–127)	91.22 ± 13.19	95 (65–125)	95.23 ± 10.69	0.002	0.016
Hip circumference (cm)	106 (90–140)	108.05 ± 11.79	107 (87–147)	108.28 ± 10.43	0.319	0.883
BMI (kg/m^2^)	27.10 (20.10–39.80)	28 ± 4.83	28.20 (18.40–44.8)	28.09 ± 4.16	0.299	0.884
Cigarette smoking (years)	2.50 (0–36)	7.75 ± 9.80	0 (0–47)	4.63 ± 10.87	0.002	0.140
Diastolic BP (mmHg)	80 (60–110)	79.59 ± 11.36	80 (55–110)	79.36 ± 11.14	0.818	0.153
Systolic BP (mmHg)	130 (95–170)	128.38 ± 17.06	140 (100–210)	141.60 ± 21.16	<0.001	<0.001
FPG (mmol/L)	5.20 (4.30–8)	5.32 ± 0.63	5.60 (3.70–12.10)	5.88 ± 1.19	<0.001	<0.001
HbA1c (%)	4.90 (4.40–6.80)	5 ± 0.50	5.10 (4.10–8.80)	5.38 ± 0.78	<0.001	<0.001
Uric acid (μmol/L)	249 (105–432)	256.68 ± 67.31	267 (136–685)	282.72 ± 77.79	0.023	0.016
Creatinine (μmL/L)	73 (47–96)	72.32 ± 11.58	76 (48–146)	79.22 ± 18.08	0.027	0.003
eGFR (mL/min/1.73 m^2^)	81 (56–113)	83.25 ± 14.20	68 (28–102)	69.23 ± 16.97	<0.001	< 0.001
ESR (mm/h)	12 (1–94)	11.75 ± 11.46	18 (2–66)	18.21 ± 9.60	<0.001	< 0.001
hsCRP (mg/L)	1.50 (0.10–94)	2.17 ± 2.56	1.60 (0–64)	2.49 ± 2.56	0.282	0.554
Total cholesterol (mmol/L)	5.90 (4.30–9.30)	6.08 ± 1.07	5.60 (2.70–8.80)	5.62 ± 1.09	0.036	0.328
LDL (mmol/L)	3.60 (2.10–6.90)	3.82 ± 0.96	3.40 (1.30–6.60)	3.44 ± 0.98	0.051	0.491
Triglycerides (mmol/L)	1.20 (0.50–3.50)	1.38 ± 0.61	1.50 (0.30–3.80)	1.46 ± 0.62	0.238	0.490
HDL (mmol/L)	1.50 (0.90–2.90)	1.51 ± 0.36	1.40 (0.70–2.60)	1.47 ± 0.35	0.363	0.477
Non-HDL (mmol/L)	4.30 (2.60–7.90)	4.58 ± 1.09	4.10 (1.40–7)	4.42 ± 3.32	0.059	0.697
Atherosclerosis index	2.56 (1.10–5.75)	2.66 ± 0.91	2.30 (1–4.90)	2.43 ± 0.77	0.283	0.052
oxLDL (pg/mL)	16.40 (3.80–154.60)	30.69 ± 41.42	11.32 (3.40–202.8)	20.33 ± 32.85	0.414	0.269
6MWT (m)	508 (315–612)	499 ± 79.13	371 (68–600)	360 ± 121	< 0.001	< 0.001
IPAQ total (MET/min/week)	1506 (0–13878)	2762.15 ± 3374	735 (0–7812)	1288.40 ± 1365	0.058	0.004
Vigorous physical activity (MET/min/week)	0 (0–8640)	1076.36 ± 2293.04	0 (0–960)	147.79 ± 307	0.211	0.003
Moderate physical activity (MET/min/week)	360 (0–5040)	771.51 ± 1102.89	240 (0–5040)	523 ± 851	0.299	0.232
Walking (MET/min/week)	594 (0–4158)	961.54 ± 1026.01	396 (0–3465)	618 ± 698	0.167	0.060
Sedentary activity (min/day)	180 (30–480)	217 ± 110	240 (30–480)	239 ± 108	0.417	0.353

Abbreviations: BMI—body mass index; BP—blood pressure; eGFR—estimated glomerular filtration rate; ESR—erythrocyte sedimentation rate; FPG—fasting plasma glucose; HbA1c—glycated haemoglobin; HDL—high-density lipoprotein; hsCRP—high-sensitivity C-reactive protein; IPAQ—International Physical Activity Questionnaire; LDL—low-density lipoprotein; MET—metabolic equivalent of task; oxLDL—oxidative modification of low-density lipoprotein; PWV—pulse wave velocity; SCORE 2/SCORE 2OP—systematic coronary risk evaluation, age 40–69 years/SCORE2-OP older persons, age 70–89 years; SD—standard deviation; 6MWT—6 min walk test; VAS—visual analog scale; WHR—waist-hip ratio. Statistical significance (*p*) was calculated with Student’s *t*-test and Mann–Whitney test with *p* ≤ 0.05 considered significant.

## Data Availability

Further information concerning the present study is available from the corresponding author upon reasonable formal request.
